# FSTL1 as a Potential Mediator of Exercise-Induced Cardioprotection in Post-Myocardial Infarction Rats

**DOI:** 10.1038/srep32424

**Published:** 2016-08-26

**Authors:** Yue Xi, Da-Wei Gong, Zhenjun Tian

**Affiliations:** 1 Institute of Sports and Exercise Biology, Shaanxi Normal University, Xi’an, Shaanxi, 710119, P. R. China; 2 Division of Endocrinology, Diabetes and Nutrition, Department of Medicine, University of Maryland School of Medicine, Baltimore, MD 21201, USA; 3 VA Research Service, Geriatric Research, Education and Clinical Center, Baltimore Veterans Administration Medical Center, Baltimore, MD 21201, USA.

## Abstract

Exercise training has been reported to ameliorate heart dysfunction in both humans and animals after myocardial infarction (MI), but the underlying mechanisms are poorly understood. Follistatin-like1 (FSTL1) is a cardioprotective factor against ischemic injury and is induced in cardiomyocytes and skeletal muscle in ischemic and hypoxic conditions. To test the hypothesis that FSTL1 may be a molecular link between exercise and improved heart function post MI, we subjected MI-rats, induced by left coronary artery ligation, to two modes of exercise: intermittent aerobic exercise (IAE) or mechanical vibration training (MVT), for four weeks and examined the relevance of FSTL1 to exercise-mediated cardiac effects. Exercise improved the functional performance, reduced fibrosis of MI-hearts and induced FSTL1 expression, the TGFβ-Smad2/3 signaling and angiogenesis in myocardium. In gastrocnemius, exercise increased the cross-sectional area of myocytes and FSTL1 expression. Importantly, exercise increased circulating FSTL1 levels, which were positively correlated with the skeletal muscle FSTL1 expression and negatively correlated with heart fibrosis. Overall, the IAE was more effective than that of MVT in cardioprotection. Finally, exogenous FSTL1 administration directly improved angiogenesis as well as functionality of post-MI hearts. Taken together, we have demonstrated that FSTL1 is a potential mediator of exercise-induced cardioprotection in post-MI rats.

Myocardial infarction (MI) is a leading cause of mortality and morbidity in the world^1^,^2^,^3^. Pathologically, MI results in immediate tissue damage due to myocardial ischemia, followed by biochemical changes triggered by reperfusion and pathological remodeling, leading to left ventricular (LV) heart failure and mortality^4^,^5^. However, despite greater understanding of the pathological processes of MI and the use of pharmacological interventions made in recent decades, post-MI mortality remains high; a 5-year survival rate is about 66.70%^6^,^7^. Therefore, novel interventional strategies to prevent ischemia/reperfusion (I/R) injury and pathological remodeling are called for to improve the post-MI survival rate.

Besides pharmacological interventions, exercise has shown cardioprotective effects against I/R injury and facilitates post-MI recovery. But how exercise mediates this beneficial effect is not well understood. One possible explanation is that skeletal muscle secretes some heart-protective factors^8^,^9^ and MI results in muscle atrophy and decrease in secretion of those factors. Conversely, exercise would counteract the muscle atrophy^10^ and hence, improve post-MI recovery. Moreover, exercise may act directly on the myocardium^11^,^12^,^13^ to improve the microenvironment of infarcted hearts.

Recently, the significance of follistatin-like1 (FSTL1), an angiogenic factor^14^, in cardiovascular system has been increasingly recognized^15^,^16^,^17^. Mice with cardiac-specific *fstl1* knock-out (cFstl1-KO) develop cardiac hypertrophy and ventricular dysfunction in response to transverse aortic constriction (TAC)^18^. FSTL1 is reported to suppress cardiac hypertrophy caused by pressure overload^18^ and to improve endothelial cells (EC) and vascular remodeling in hypoxic-ischemic regions^14^. Intriguingly, FSTL1 is secreted from both skeletal muscle^19^ and myocardium^15^,^20^ and the muscle-derived FSTL1 can function as an endocrine hormone to modulate vascular remodeling in response to wire-induced artery injury^21^. However, whether and how FSTL1 is regulated by exercise has not been studied. Different modes of exercise have been reported to affect post-MI recovery differently: Intermittent aerobic exercise (IAE) is effective in diminishing pathological myocardial transformation in the post-infarction failing rat heart^22^, increasing peak oxygen uptake^23^ and improving functional capacity and life quality in patients with chronic heart failure (CHF)^24^, whereas mechanical vibration training (MVT) accelerates the reperfusion of vessels^25^ and elevates circulating levels of angiogenic regulators such as VEGF and MMP-2/9 in humans^26^. This study aimed to address the questions of whether FSTL1 is involved in exercise-mediated protection of post-MI hearts and which exercise mode, IAE or MVT, is more effective in cardioprotection. We found that exercise stimulated FSTL1 expression in skeletal muscle and myocardium after acute MI, concurrently with enhanced TGFβ-Smad2/3 signaling, increased myocardium angiogenesis and improved heart functional performance. Significantly, IAE was more effective than MVT in cardioprotection after MI.

## Results

### Exercise mitigates heart dysfunction and reduces heart fibrosis in MI rats

**Figure 1:**
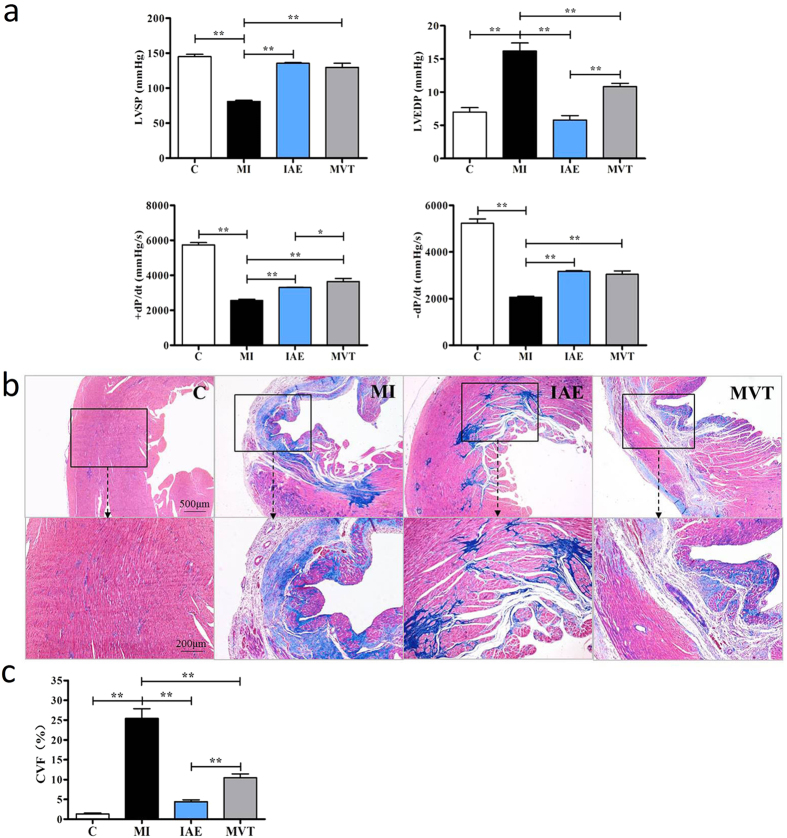
Exercise improves heart function and reduces myocardial fibrosis. (**a**) Rats were subjected to sham operation (C) or myocardial infarction (MI). The MI rats were trained without or with intermittent aerobic exercise (IAE) or mechanical vibration training (MVT) for 4 weeks when heart hemodynamics were measured. LVSP, left ventricular systolic pressure; LVEDP, left ventricular end-diastolic pressure; ±dP/dt, maximum pressure increasing/decreasing rate. (**b**) Masson staining of heart tissue section for fibrosis evaluation. Collagen is stained blue, indicating fibrosis. The lower image is an amplification of the upper black box. (**c**) Quantification of collagen volume of fraction (CVF). Data are mean + SE (n = 6); *p < 0.05; **p < 0.01.

We studied the effect of exercise on post-MI hearts in four groups of animals: MI control (MI) by left coronary artery (LAD) ligation, MI with exercises (IAE vs MVT), and sham operation control (C). As expected, heart functional parameters of the left ventricular systolic pressure (LVSP) and the contractility index, absolute value of ±dP/dt(max), in the group of MI were greatly decreased whereas the left ventricular end-diastolic pressure (LVEDP) was increased, compared to the control group, indicating a heart dysfunction (Fig. 1a). Significantly, exercise fully or partially restored these indexes, and IAE appeared more effective in lowering LVEDP than MVT (Fig. 1a). Thus, exercise improved the functionalities of the post-MI heart. Masson staining indicated strong blue collagen staining in myocardium of the MI group (Fig. 1b). Both the IAE and MVT modes of exercise reduced the staining area, but the degree of reduction is greater in the IAE group (Fig. 1c). These results demonstrate that exercise mitigated dysfunction in the post-MI heart and reduced heart fibrosis, with IAE being more effective in these respects.

### Exercise induces myocardial FSTL1 expression and angiogenesis and activates TGFβ-Smad2/3 signaling

**Figure 2:**
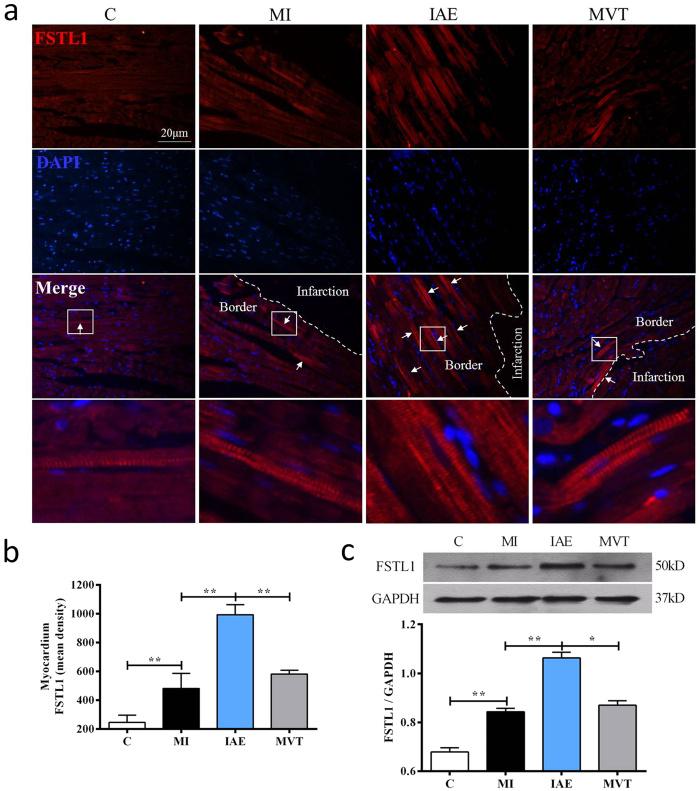
Effect of exercise on myocardium expression of FSTL1. (**a**) Immunohistochemistry (IHC) of FSTL1 in cardiomyocytes. IHC staining (upper row) of FSTL1 in groups of control (C), MI, MI with IAE and MI with MVT, was counterstained with DAPI (blue) for nuclei (second raw). The third row is a merged image of FSTL1 with DAPI, indicating that FSTL1 (representatively indicated by small arrows in a small square) is expressed in the non-infarction boarder area of myocardium. The fourth row is the amplification of the small square area. (**b**) Quantification of the immunofluorescent staining. (**c**) Western blot analysis of FSTL1 and quantification (lower) with GAPGH as the loading control. Data are mean + SE (n = 6); *p < 0.05; **p < 0.01.

Next, we investigated whether exercise regulates the expression of FSTL1 by immunohistochemistry (IHC). As shown in Fig. 2a, FSTL1 was detected in the cytoplasm of cardiomyocytes and appeared to be increased in the non-infarction area in the MI group. IAE further increased the staining intensity. Quantitatively, compared to the control group, MI induced FSTL1 expression by 1.96 fold, which was further induced by 4.04 fold by IAE but not by MVT (Fig. 2b). Further western analyses (Fig. 2c) of FSTL1 confirmed the IHC findings.

**Figure 3:**
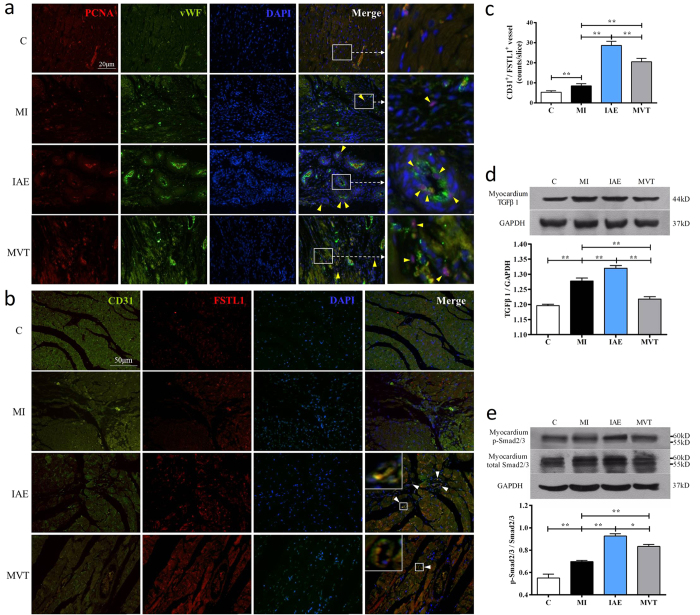
Exercise stimulates myocardium angiogenesis, induces CD31^+^/FSTL1^+^ cell proliferation and activates TGFβ-Smad2/3 signaling. (**a**) Double staining of PCNA and vWF markers, with DAPI nuclei staining in myocardium of the sham-op control (C), MI, MI with IAE and MI with MVT. Arrows indicate the PCNA^+^/vWF^+^ cells and the farthest right image is an amplification of the white box within the merged image. More vessel-like structures are seen in IAE and MVT. (**b**) Co-staining of CD31^+^/FSTL1^+^ shows that endothelial cells form capillary-like structure indicated by arrows in IAE and MVT. Inserts are amplification of white boxes. (**c**) Quantification of the number of CD31^+^/FSTL1^+^ capillaries per slice. (**d**) Western analysis and quantification of TGFβ1. (**e**) Western blot analysis and quantification of Smad2/3 and phosphorylated Smad2/3 (p-Smad). Data are mean + SE (n = 6); *p < 0.05; **p < 0.01.

FSTL1 is a known angiogenic factor^14^,^27^ and induction of FSTL1 would promote angiogenesis. Hence, we examined endothelial cell proliferation by co-staining of PCNA^+^, a cell proliferation marker and vWF^+^, an endothelial cell marker. Compared with the control group, MI induced more PCNA^+^/vWF^+^ cells, whose number was increased and distribution expanded by exercise (Fig 3a). Notably in the IAE group, some double stained cells appeared to form new small vessel-like structures whereas in the MVT group, the double-stained cells were more diffuse (Fig. 3a). Further co-staining of FSTL1 with CD31, an endothelial cell marker, revealed a significant increase in the number of CD31^+^/FSTL1^+^ vessels (8.50 ± 1.12 counts) in the MI group, compared to the control (Fig. 3b,c). The number of double stained vessels was further increased in the IAE (28.67 ± 2.06 counts) and MVT groups (20.50 ± 1.69 counts).

We speculated that FSTL1 might act like FST through the TGFβ-Smad2/3 signaling pathway^28^ to induce angiogenesis^29^ and investigated the effect of exercise on the pathway. As a result, TGFβ1 protein expression was found to be up-regulated after MI, and further induced by IAE (Fig. 3d), but MVT had no effect on the protein expression. Downstreamly, Smad2/3 levels were significantly increased in MI and further up-regulated in IAE and MVT, but the former was more effective than MVT (Fig. 3e). Together, these results show that exercise, especially IAE, induced FSTL1 expression in myocardium of the infarct heart, promoted angiogenesis and enhanced angiogenesis-related signaling.

### Exercise reverses the skeletal muscle atrophy and increases the FSTL1 expression in skeletal muscle and serum after MI

**Figure 4:**
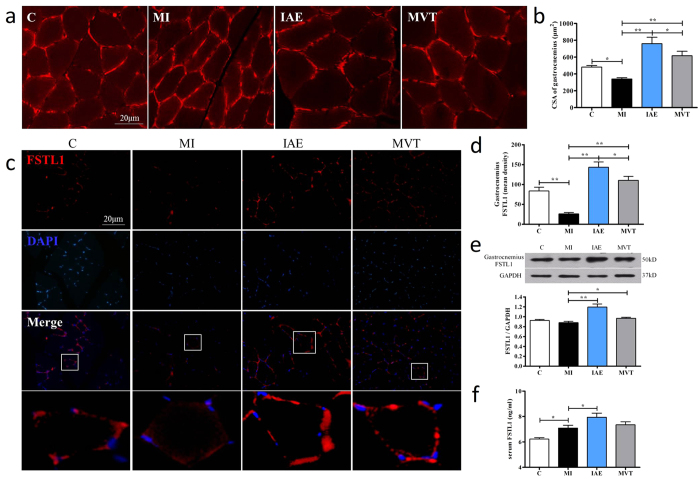
Exercise reverses MI-induced myoatrophy and increases muscle FSTL1 expression and blood FSTL1 levels. (**a**) Staining of gastrocnemius myocyte membrane by DiI in sham-op control (C), MI and MI with exercise (IAE or MVT) for 4 weeks, with quantification (**b**) of cell cross-sectional area (CSA) of the average of 100 cells. Immunofluorescent staining (**c**) and quantification (**d**) and Western analysis (**e**) of FSTL1 in gastrocnemius muscle. Stronger staining is observed in IAE and MVT groups. f: Serum levels of FSTL1 in the groups of rats. Data are mean + SE (n = 6); *p < 0.05; **p < 0.01.

Skeletal muscle is a main source of the circulating FSTL1^19^,^30^, and we next investigated the effect of exercise on skeletal muscle mass and FSTL1 expression. We labeled the cell membrane by using derivatives of indocarbocyanine iodide (DiI) to measure changes of the gastrocnemius cell cross-sectional area (CSA) as a representative parameter for skeletal muscle mass^31^,^32^. In consistent with previous reports^33^,^34^, compared with the control group (481.73 ± 18.60 μm^2^/cell), CSA was significantly decreased after MI (340.50 ± 13.87 μm^2^/cell, p < 0.01), indicating myoatrophy. After 4 weeks of exercise, CSA increased by 2.23 fold and 1.81 fold in IAE and MVT groups (both p < 0.01 vs. the MI, Fig. 4a,b), respectively.

**Figure 5:**
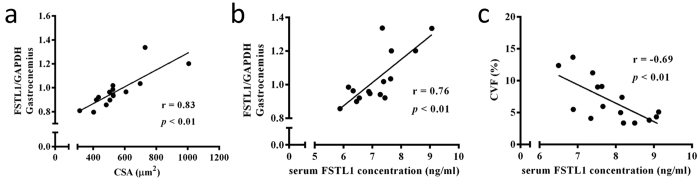
Correlation of serum FSTL1 levels with muscle FSTL1 expression and with heart fibrosis. Positive correlations of skeletal muscle FSTL1 expression with gastrocnemius myocytes cross-section area (CSA) (**a**) and with serum FSTL1 levels (**b**). (**c**) Negative correlation of serum FSTL1 with heart collagen volume of fraction (CVF). Pearson correlations were conducted for the above analyses (n = 15, pooled samples of C, MI, IAE and MVT).

IHC showed that FSTL1^+^ myocytes were sporadically detected in the control group, and were sparse in MI (p < 0.01). IAE and MVT significantly increased the number and intensity of the FSTL1^+^ cells (Fig. 4c,d). Western quantification showed that IAE and MVT significantly increased the FSTL1 expression by 36% and 10% respectively vs. the MI group (Fig. 4e). Furthermore, we measured circulating FSTL1 levels. Compared to the control group, circulating FSTL1 levels were increased post-MI (Control 6.22 ± 0.11 vs. MI 7.09 ± 0.22 ng/ml; p < 0.05), which were further increased by IAE (7.94 ± 0.33 ng/ml), but not by MVT (7.35 ± 0.24 ng/ml, Fig. 4f). Correlation analyses revealed significant positive correlations between the gastrocnemius CSA and the skeletal muscle FSTL1 expression (r = 0.83, p < 0.01; Fig. 5a), and between the skeletal muscle FSTL1 expression and the serum FSTL1 concentration (r = 0.76, p < 0.01; Fig. 5b), with a negative correlation between the serum FSTL1 and the heart collagen volume fraction (CVF) (r = −0.69, Fig. 5c). Collectively, these findings show a significant increase of gastrocnemius CSA, skeletal muscle FSTL1 expression and serum FSTL1 levels, and a remarkable correlation of the expression of skeletal muscle FSTL1 with the level of serum FSTL1.

### Exogenous FSTL1 improves angiogenesis, enhances TGFβ-Smad2/3 signaling and heart function after MI

**Figure 6:**
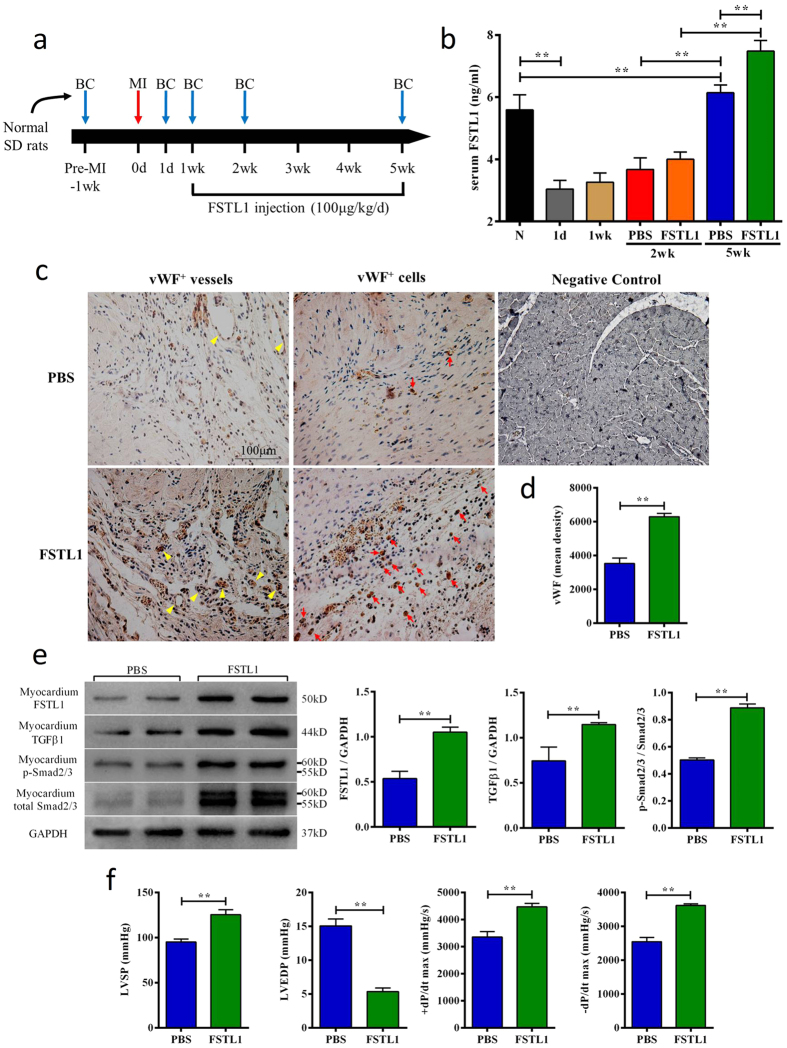
Exogenous FSTL1 directly stimulates heart angiogenesis, activates TGFβ-Smad2/3 signaling and improves functional performance. (**a**) Scheme of the animal study. Normal rats underwent MI by LAD ligation on Day 0. Blood collection (BC) took places at the indicated time points for FSTL1 measurements. Recombinant FSTL1 was administered one week post-MI and continued daily for 4 weeks. Immunohistochemistry and functional assessment were conducted at the end of week 5. (**b**) Changes of blood FSTL1 levels of rats treated with or without FSTL1 during the animal protocol. (**c**,**d**) Immunohistochemistry staining shows more vWF^+^ vascular structure (yellow arrow) and vWF^+^ cells (red arrow) in rats treated with FSTL1 than the PBS control, and quantification of mean optical density per view. (**e**) Enhanced TGFβ1/p-Smad2/3 signaling in myocardium of rats treated with FSTL1 vs. PBS control, as analyzed by Western blot and quantification. (**f**) Functional assessment of hemodynamic parameters in rats treated with PBS or FSTL1. LVSP: left ventricular systolic pressure; LVEDP: left ventricular end-diastolic pressure.; and ±dP/dt: maximum pressure increasing/decreasing rate. Data are expressed as mean + SE (n = 6); *p < 0.05; **p < 0.01.

To determine whether FSTL1 exerts a direct effect on post-MI hearts, we administrated FSTL1 at the dosage of 100 μg/kg body weight/day^17^ from 1wk to 5wk post-MI and serum FSTL1 was measured at several time points as illustrated in Fig. 6a. Compared to the pre-MI level, serum FSTL1 decreased by 45.57% on day 1 after MI and remained lower during the acute phase of MI (1 day-1week) and gradually increased at week 2 and week 5. Administration of FSTL1 from week 1 resulted in a small increase at week 2 and a statistically significant increase at week 5, compared to the control group. Serum FSTL1 levels were elevated in the FSTL1 administration group (Fig. 6b). IHC of myocardium at week 5 revealed more vWF^+^ cells and small vessels in the FSTL1 group than in the PBS control group (Fig. 6c,d). In addition, at the protein level, exogenous FSTL1 administration significantly up-regulated heart FSTL1 and TGFβ1 protein expressions at week 5 by 95.81% and 54.14% vs. the PBS control, respectively. Interestingly, Smad2/3 and p-Smad2/3 expressions were also increased in FSTL1 group, while the p-Smad2/3 vs. Smad2/3 ratio had risen about 76.70% (p < 0.01 vs. the control group, Fig. 6e). Additionally, exogenous FSTL1 administration enhanced signaling pathways of Akt, Erk1/2 and AMPK but not of Smad1/5/8 in MI hearts (Fig. S2). Importantly, FSTL1-treated animals showed a significant improvement of heart function with an increase of LVSP by 31.71%, +dP/dt(max) by 33.17%, −dP/dt(max) by 41.97% and a decrease of LVEDP by 64.49% at week 5 (p < 0.01, Fig. 6f). These results demonstrate that FSTL1 exerts direct cardioprotective action by promoting angiogenesis, increasing FSTL1 protein content and activating TGFβ-Smad2/3 signaling in the post-MI heart, all of which mitigate heart dysfunction.

## Discussion

In this study, we have replicated exercise’s cardioprotective effect in post-MI hearts^35^,^36^ and demonstrated that exercise can significantly induce skeletal muscle and cardiac expression of FSTL1 and increase its circulation levels. Importantly, the level of skeletal muscle FSTL1 expression is significantly correlated with its circulating levels and associated with heart functional performance, providing a piece of evidence that exercise may exert its cardioprotection through increased production and secretion of FSTL1 in the skeletal muscle and myocardium.

Which tissue of FSTL1 expression (skeletal muscle or myocardium) is crucial to the exercise-mediated cardiac benefits is an intriguing question but difficult to answer for the time being. Cardiac FSTL1 expression is increased in response to heart ischemic injury^17^,^20^. Mice with cardiomyocyte-specific deletion of FSTL1 are susceptible to, whereas mice with FSTL1 overexpression are resistant to, heart dysfunction following transverse aortic constriction (TAC)^18^. Thus, myocardial FSTL1 appears to be cardioprotective again heart injury. Recently, Wei *et al*. has reported that epicardial FSTL1 can promote immature cardiac myocyte proliferation and diminishes infarct size post-MI^15^, and that myocardial FSTL1 is insufficient for long-term recovery from MI. Nevertheless, this study does not negate a possible significance of endogenous myocardial FSTL1 in cardioprotection. Angiogenesis is an essential part of heart repair post-MI; it helps to salvage ischemic myocardium at the early stages and is also essential for long-term left ventricular remodeling to prevent the transition to heart failure^37^,^38^. Angiogenesis by TGFβ is known to be partly mediated by Smad2/3 activation^39^,^40^. We found that the increased myocardial FSTL1 expression was associated with enhanced TGFβ-Smad2/3 signaling and angiogenesis, suggesting that FSTL1 may exert its cardiac benefits through angiogenesis.

FSTL1 can activate and increase the expression of TGFβ^41^. In the present study, we observed that TGFβ, total Smad2/3 and p-Smad2/3 protein levels were increased by exogenous FSTL1 administration. This finding is consistent with prior studies^42^,^43^ where co-regulations among TGFβ and total Smad2/3 protein expression p-Smad2/3 level are reported, suggesting a possible autoregulation mechanism^42^. Interestingly, Akt, Erk1/2 and AMPK signaling pathways were also enhanced in myocardium of rats treated with FSTL1 after MI, and Smad1/5/8 was significantly activated after exercise (Figs S1 and S2). These results suggested that multiple signaling mechanisms may participate in the FSTL1-mediated cardioprotection in MI, which deserves further elucidation.

The role of skeletal muscle in cardioprotection in MI animal models has been previously recognized. For example, a brief pre-MI ischemic insult of skeletal muscle is reported to decrease infarct size^43^,^44^,^45^ and chronic ischemia of skeletal muscle can increase left ventricle coronary vessel density^46^. These results suggest that ischemic muscle may exert its cardioprotection through some neurohumoral mechanism^47^. However, such a factor(s) has not been fully defined. Since exercise has muscle ischemia-like effects through hypoxia^48^, it may exert cardiac protective action through a similar mechanism to ischemia. Exercise is a strong stimulator of muscle growth, metabolism and endocrine function^49^. Thus, both the increased muscle mass and ischemia-mimic effect through exercise may contribute to the increased circulating levels of FSTL1. The functional significance of circulating FSTL1 was demonstrated by our animal study where exogenous FSTL1 administration activated the TGFβ-Smad2/3 signaling, promoted angiogenesis and ameliorated heart dysfunctions (Fig. 6).

FSTL1 has been increasingly recognized as a potent cardiac protection factor^15^,^16^,^17^. Whether exercise increases the heart FSTL1 expression through a peripheral, neuronal or cardiac mechanism will be a subject of future study. Interestingly, we found that exogenous FSTL1 administration increased FSTL1 expression (Fig. 6), suggesting that there might be a positive feedback regulation of circulating FSTL1 on myocardial FSTL1, probably through modulating the local microenvironment, e.g. angiogenesis.

In this study, we trained post-MI rats with IAE and MVT out of the consideration that the exercise mode and intensity are reported to affect the endocrine function of skeletal muscle and post-MI cardiac outcome differently^50^,^51^,^52^. For instance, twice a week IAE for 12 weeks at the intensity of 4 × 4 minute intervals at 85–95% of peak heart rate can diminish myocardial damage through increasing peak oxygen uptake^23^ and improving heart functional capacity and quality of life. LVEDP, left ventricular mass/body mass ratio (LVM:BM) and total CVF were decreased while LVSP and +dP/dt(max) were increased by aerobic interval training (40 min/day with 8 min of warm-up at 10 m/min and exercise at 15 m/min 4 × 4 min interspersed with 4 × 4 min at 23 m/min) in rats with chronic heart failure^53^. These results are consistent with our findings. Mechanical vibration mode by using the vibration platform with a peak-to-peak amplitude of 4 mm and a frequency of 30 Hz has been reported to improve microvessel circulation^25^,^26^. But no comparative studies of IAE vs. MVT have been reported previously. Our study shows that IAE has more protective benefits for post-MI hearts than MVT, resulting in more myocardial angiogenesis, less fibrosis and improved heart functionality. These results may be instructive for rehabilitation of post-MI patients. Importantly, IAE increases more skeletal muscle and myocardial expression of FSTL1, providing another piece of evidence that FSTL1 may be a mediator of exercise’s cardiac benefits.

In conclusion, we have demonstrated that exercise may exert its beneficial effect on post-MI hearts through the induction of cardiac and muscle FSTL1 expression. Importantly, exercise-regulated skeletal muscle FSTL1 expression is highly correlated with serum levels as well as increased heart functional performance and decreased heart fibrosis. Moreover, FSTL1’s signaling pathway is activated during exercise and exogenous FSTL1 administration. Thus, the induction of FSTL1 provides an explanation of exercise-mediated cardioprotection in post-MI hearts. Future studies using muscle- or heart-specific FSTL1 over-expression or knockout models will help to address the question of the relative significance of FSTL1 in cardioprotection between the two tissues by exercise.

## Materials and Methods

### Animals

Male Sprague-Dawley rats(200 ± 20 g, 8-weeks old)were from the Laboratory Animal Centre of Xi’an Jiaotong University. Animal studies were performed in accordance with the “Guiding principles for research involving animals and human beings”^54^. All experimental protocols were approved by the Review Committee for the Use of Human or Animal Subjects of Shaanxi Normal University.

### MI Surgical Procedure

Rats were anesthetized by pentobarbital sodium (30 mg/kg body weight). MI was induced by using the established method of left anterior descending (LAD) coronary artery ligation^55^. In brief, the coronary artery was ligated 2.0 mm from its origin using a 6.0 suture silk. An ST-segment elevation was observed by electrocardiogram after suture. Sham-operation control rats underwent the operation procedure without LAD ligation.

### Exercise Protocol

One week after infarction, rats underwent one week of adaptive training before four weeks of normal exercise. The intermittent aerobic exercise (IAE) group was subjected to a training protocol using a motorized rodent treadmill (DSPT-202, Li Tai Technology, Hangzhou, China). For daily training, animals would first start with a warm-up for 10 min and then the exercise alternated between 7 min at 25 meter/min (85–90% VO_2_max) and 3 min at 15 meter/min (50–60% VO_2_max) for 1 hr. This protocol was performed once a day, 5 days a week for 4 weeks^56^. The mechanical vibration training (MVT) group was subjected to a custom-made small animal mechanical vibration platform. Rats were vibrated in the shaker at a frequency of 25 Hz with amplitude of 2 mm for everyday training. This protocol was performed for twice a day, 38 mins per day and 5 days per week for 4 weeks^57^. No rats died by the end of these two protocols.

### Hemodynamic Measurement

At the end of the 4 weeks of training or ad-lib activity, rats were anesthetized with pentobarbital sodium (30 mg/kg body weight). A pressure transducer was inserted retrograde from the right carotid artery to the LV cavity, and intraventricular catheter recordings were performed by using Powerlab 8/30 (ML 870, AD Instruments, Castle Hill, Australia) to evaluate cardiac function. LV systolic pressure (LVSP, mmHg), LV end-diastolic pressure (LVEDP, mmHg), heart rate, and maximal positive and negative first derivative of LV pressure (±dP/dt max) were measured and calculated.

### Tissue Histology

Heart and gastrocnemius tissue samples were fixed in ice-cold 4% paraformaldehyde for 24–48 hrs, dehydrated in a concentration gradient of ethanol, embedded in paraffin and sectioned (5 μm) for histopathologic examination. To evaluate the degree of skeletal muscle atrophy, the gastrocnemius slices were stained with DiI (Sigma-Aldrich, St. Louis, MO, USA), and the cross-sectional area of skeletal muscle cells was calculated. To evaluate the degree of myocardium fibrosis, heart tissue slices were stained with Masson’s trichrome, and the collagen volume fraction (CVF) was measured.

### Western Blotting

Tissues from the LV infarct border area (5 mm) and gastrocnemius were homogenized. Total proteins extraction and SDS-PAGE were performed as described before^58^. The dilution of primary antibodies as follows: FSTL1 (1:1000, GeneTex, Irvine, CA, USA), TGFβ1 (1:500, Bioworld Technology, St. Louis, MN, USA), Smad2/3 (1:1000, Bioworld), p-Smad2/3 (1:500, Bioworld), Akt (1:2000, Cell Signaling, Danvers, MA, USA), p-Akt (1:2000, Cell Signaling), Erk1/2 (1:500, Signalway Antibody, College Park, MD, USA), p-Erk1/2 (1:500, Signalway Antibody), AMPKα1 (1:500, Signalway Antibody), p-AMPKα1 (1:500, Signalway Antibody), Smad1/5/8(1:500, Signalway Antibody), p-Smad1/5/8(1:1000, Cell Signaling) and GAPDH (1:10000, Bioworld). Following incubating with horseradish peroxidase (HRP)-conjugated secondary antibody (1:5000 dilution, Jackson ImmunoResearch, West Grove, PA, USA), protein bands were subsequently developed with enhanced chemiluminescence. Western quantification was performed by Image Processing and Analysis in Java 1.48 (Wayne Rasband, National Institutes of Health, USA).

### Immunohistochemistry

Heart sections were incubated with rabbit polyclonal antibody vWF (1:50 dilution, Millipore) over night at 4 °C after deparaffination, 3% H_2_O_2_ treatment and antigen retrieval. HRP-conjugated goat anti-rabbit IgG (R&D, Minneapolis, MN 55413) was used as a secondary antibody and diaminobenzidine (R&D) for color development. Nuclei were stained by hematoxylin. For immunofluorescent staining, sections were incubated with primary antibody over night at 4 °C after deparaffination and antigen retrieval. The dilutions of primary antibodies were as follows: rabbit polyclonal antibody FSTL1 (1:100 dilution, GeneTex), rabbit polyclonal antibody vWF (1:50 dilution, Millipore), mouse monoclonal antibody CD31 (1:25 dilution, GeneTex) and mouse monoclonal antibody PCNA (1:50 dilution, Cell Signaling). TRITC/FITC-conjugated goat anti-rabbit/mouse IgG (1:100 dilution, Jackson ImmunoResearch) were used as secondary antibodies. Nuclei were stained by 4′6-diamidino-2-phenylindole (DAPI) dye (1:800 dilution, Sigma, St. Louis, MO, USA). Results were observed with a fluorescence microscope (Nikon Eclipse 55i, Nikon, Tokyo, Japan).

### FSTL1 Administration and ELISA

One week post-operation, recombinant FSTL1 protein (ProSpec, East Brunswick, NJ, USA) was administrated by intraperitoneal injection at the dosage of 100 μg/kg bodyweight/day^17^ for 4 weeks. Blood was taken as indicated in Fig. 6a. ELISA was performed according to the manufacturer’s instruction (BD Biosciences).

### Statistical Analysis

Density mean of random views were calculated by Image-Pro Plus 6.0 (Media Cybernetics, Bethesda, MD, USA). All data were presented as mean ± SE in this study. Statistical analysis was performed using SPSS 17.0. One-way analysis of variance (ANOVA) was used for group comparison and the Pearson correlation method was used to assess possible pairwise relationships. p < 0.05 was considered significant. Histograms were plotted by Prism 5.01 (GraphPad Software, La Jolla, CA, USA).

## Additional Information

**How to cite this article**: Xi, Y. *et al*. FSTL1 as a Potential Mediator of Exercise-Induced Cardioprotection in Post-Myocardial Infarction Rats. *Sci. Rep.* **6**, 32424; doi: 10.1038/srep32424 (2016).

## Supplementary Material

Supplementary Information
