# Supplementary material for: FSTL1 as a Potential Mediator of Exercise-Induced Cardioprotection in Post-Myocardial Infarction Rats
Source: Sci Rep. 2016 Aug 26;6:32424. doi: 10.1038/srep32424 (PMC5000295; doi:10.1038/srep32424)
Supplement: Supplementary Information [file srep32424-s1.pdf]

Supplementary Materials for

FSTL1 as a Potential Mediator of Exercise-Induced Cardioprotection in Post-Myocardial Infarction Rats

Yue Xi, Da-Wei Gong, Zhenjun Tian*

*Corresponding author. E-mail: tianzj2013@hotmail.com

The Supplementary Materials includes:

Figure S1. Enhanced Akt, Erk1/2, AMPK and Smad1/5/8 signaling in myocardium of rats underwent MI and exercise.

Figure S2. Enhanced Akt, Erk1/2 and AMPK but not Smad1/5/8 signaling in myocardium of rats treated with FSTL1 vs. PBS control.

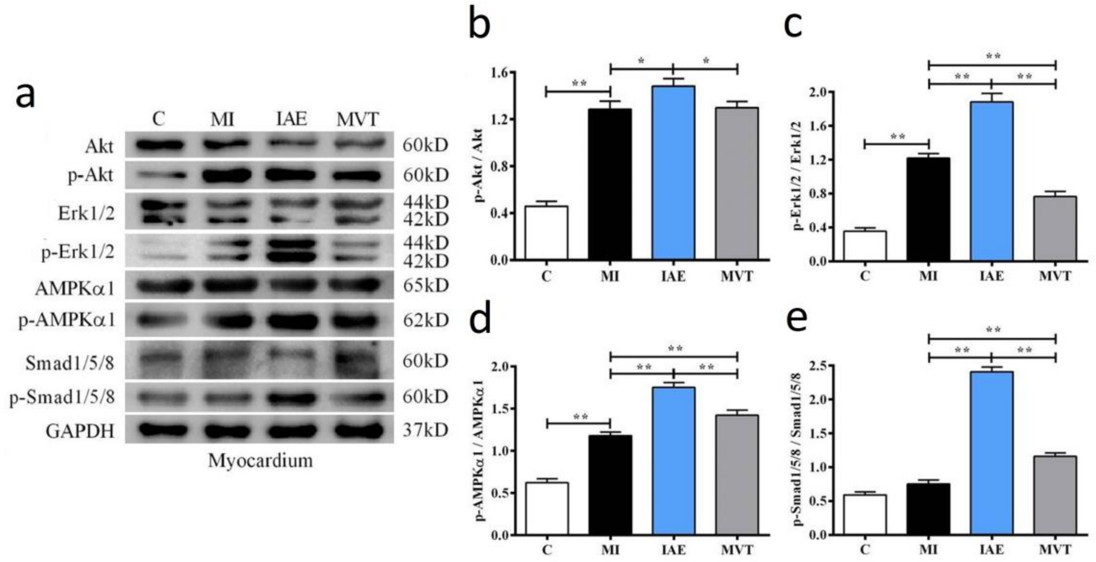

Figure S1. Enhanced Akt, Erk1/2, AMPK and Smad1/5/8 signaling in myocardium of rats underwent MI and exercise

Rats were subjected to sham operation (C) or myocardial infarction (MI). The MI rats were trained without or with intermittent aerobic exercise (IAE) or mechanical vibration training (MVT) for 4 weeks. a: Western blot results of Akt, phosphorylated Akt (p-Akt), Erk1/2, p-Erk1/2, AMPKα1, p-AMPKα1, Smad1/5/8 and p-Smad1/5/8. b-e: The quantification of Western blot analysis. Data are mean + SE (n = 6); *: $p < 0.05$; **: $p < 0.01$.

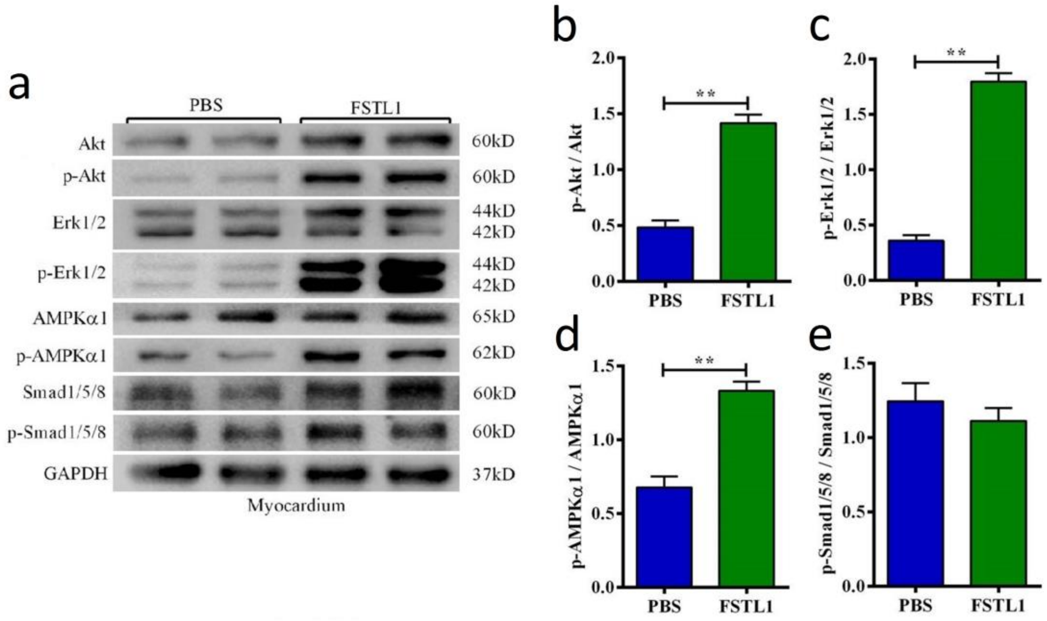

Figure S2. Enhanced Akt, Erk1/2 and AMPK but not Smad1/5/8 signaling in myocardium of rats treated with FSTL1 versus PBS control

Recombinant FSTL1 was administered one week post-MI and continued daily for 4 weeks (FSTL1), PBS injection as control (PBS). a: Western blot results of Akt, p-Akt, Erk1/2, p-Erk1/2, AMPKα1, p-AMPKα1, Smad1/5/8 and p-Smad1/5/8. b-e: The quantification of Western blot analysis. Data are expressed as mean + SE (n = 6); *: $p < 0.05$; **: $p < 0.01$.
